# Predictors of relapses in ANCA-associated small vessel vasculitis with kidney involvement

**Published:** 2014

**Authors:** Andreiana Iuliana, Stancu Simona, Avram Andreea, Taran Ludmila, Mircescu Gabriel

**Affiliations:** * Departement of Nephrology and Dialysis, "Dr. Carol Davila" Teaching Hospital of Nephrology, Bucharest, Romania; ** Departement of Internal Medicine and Nephrology, “Carol Davila” University of Medicine and Pharmacy, Bucharest, Romania

**Keywords:** ANCA vasculitis, kidney, relapse

## Abstract

**Rationale:** Almost half of the patients with anti-neutrophil cytoplasmic antibodies (ANCA) associated small vessel vasculitis relapse and their characteristics are still to be defined

**Objective:** We aimed to evaluate the relapse rate and its determinants in a cohort of patients with ANCA associated vasculitis with severe kidney involvement.

**Methods and results:** This is a retrospective study which included 100 patients consecutively admitted in a Nephrology Department with crescentic pauci-immune glomerulonephritis diagnosed by kidney biopsy. ANCAs were assessed by capture ELISA or indirect immunofluorescence (IFI). Patients were followed for a median period of 3.2 [0.1; 5.5] years. The median age was 61.6 years. The clinical condition at presentation was severe (median BVAS 16 and BVAS over 21 in one quarter of patients), mostly because of general, kidney and lung scores. Median creatinine was 5.7 mg/dL and 17% of the patients needed temporary dialysis. Eight patients relapsed (13.8%): one in the lung and seven in the kidney. The median time to relapse was 11.3 [9.2; 19.9] months. None of the investigated parameters allowed for differentiating patients who relapsed from those who did not, except higher hematuria in those who relapsed.

**Discussion:** In our patients with ANCA vasculitis and severe kidney involvement, the relapse rate is low and hematuria but not ANCA specificity or clinical presentation allows the prediction of relapse.

## Introduction

Anti-neutrophil cytoplasmic antibodies (ANCA) associated small vessel vasculitis are rare but severe autoimmune diseases which share as central pathogenic element the formation of antibodies against myeloperoxidase (MPO-ANCA) and proteinase 3 (PR3-ANCA). There are four clinical entities in this group: microscopic polyangiitis (MPA) which is frequently associated with MPO-ANCA, granulomatosis associated with polyangiitis (GPA, formerly known as Wegener’s granulomatosis) which is frequently associated with PR3-ANCA, eosinophilic granulomatosis associated with polyangiitis (also known as Churg Strauss syndrome) and the single organ limited vasculitis [**1**].

Kidney involvement, e.g. crescentic pauci-immune glomerulonephritis with acute kidney injury, is frequent (70-80% of cases). Accordingly, nephrologists are usually part of the multidisciplinary team caring patients with ANCA-vasculitis.

The natural evolution of ANCA vasculitis is rapidly progressive, usually to death or renal replacement therapy. Half of the patients die in the first six months and 90% of them in the first two years [**2**].

The therapy with steroids and immunosuppressive drugs dramatically improved outcome, but between 10 and 20% of patients don’t respond to treatment, relapses appears during and after the treatment, it can be a source of severe complications (infections, cancer, cardiovascular morbidity and infertility) [**3**-**6**]. In consequence, patients’ survival remained substantially lower than expected in the general population [**7**].

Between 19 and 56% of the patients have at least one relapse during evolution, usually in the first five years [**6**,**8**,**9**,**10**] and relapses were associated with poorer survival [**11**]. On the other hand, more than half of the patients never relapse and in these patients the maintenance therapy is useless.

Unfortunately, the patients which relapse are insufficiently characterized yet. It appears that patients with GPA, PR3-ANCA positive patients and those with respiratory involvement are more prone for relapse [**4**,**6**,**12**-**15**].

On the other hand, relapses seemed to be less frequent in patients with kidney involvement but they were associated with accelerated loss of kidney function [**16**].

Considering these observations, we aimed to evaluate the frequency and the determinants of relapses in a cohort of patients with severe vasculitis admitted in a Nephrology clinic. As far as we know, this is the first report which adressing this issue in Romania.

## Material and Methods

**Patients**

This is a cohort study on all adult patients consecutively admitted, diagnosed by kidney biopsy and followed in the “Dr Carol Davila” Teaching Hospital of Nephrology, Bucharest. From January 2000 till January 2014, 104 patients were diagnosed with crescentic pauci-immune glomerulonephritis by kidney biopsy. The criteria for the histological diagnosis were crescent in more than 50% of examined glomeruli by light microscopy and a direct immunofluorescence assay for complement and immunoglobulins of 0 to 1+ on a scale 0 to 4+.

All subjects signed an informed consent form authorising us to use their demographic and medical data in this study.

The study was approved by the local Ethics Committee.

## Diagnosis and follow-up

ANCAs were assessed by capture PR3-ANCA and MPO–ANCA ELISA (Euroimmun™, Lübeck, Germany) or by indirect immunofluorescence (IFI) with monoclonal mouse anti-human myeloperoxidase antibodies (Dako™, Glostrup, Denmark). Because two methods were used along time to measure ANCA levels, the results were not comparable and were not included in analyses. The patients were grouped as MPO-ANCA patients (MPO-ANCA sero-positive or ANCAp pattern), PR3-ANCA patients (PR3-ANCA sero-positive or ANCAc pattern) and N-ANCA patients (ANCA sero-negative by ELISA and IFI).

Birmingham Vasculitis Activity Score version 3 (BVAS) was computed retrospectively by the same investigator and used to evaluate the severity of vasculitis [**17**].

Inflammation was assessed using serum fibrinogen, serum albumin, white blood cells and platelets number.

Kidney damage was evaluated by diuresis (oliguria was defined as an urinary volume under 800/mL/day), proteinuria in a 24 hours urine collection, hematuria (macroscopic and microscopic, red blood cell casts) and serum creatinine.

The measurements were performed with standard laboratory methods: biochemistry on an Olympus AU 400 auto-analyzer, and hematology on a MINDRAY BC 3000 auto-analyzer.

Follow-up protocol included monthly visits with clinical (BVAS) and laboratory evaluation till remission and each three months thereafter for at least 2 years.

**Definitions**

Renal response to therapy was defined as disappearance of hematuria and stable or improving serum creatinine [**18**].

Relapse was defined as reappearance of hematuria or hemoptysis accompanied by an increase in BVAS in responders.

Dialysis therapy was considered temporary when needed for less than 3 months and chronic when longer.

**Treatment**

Patients were treated using the same protocol over the whole study period: induction of remission in the first 6-9 months with methylprednisolone, 0.5-1g, 3 daily intravenous pulses and cyclophosphamide 0.5-1g/m2, one intravenous pulse each 2-4 weeks, and maintenance of remission with prednisone, 0.5 mg/body weight per day, gradually tapered to 5-7.5 mg/day in association with azathioprine 1.5-2 mg/ body weight per day, for 2 to 5 years. Plasmapheresis was occasionally performed, in case of severe hemorrhagic alveolitis.

**Study end-point**

The occurence of a relapse.

**Statistical analysis**

Categorical variables are presented as percentages and comparison test were performed using Pearson χ2 test. Continuous variables are displayed as mean and 95% confidence interval (95% CI) or median and quartiles [1; 3], according to their distribution. Comparisons were done with Welch-ANOVA, Mann-Whitney and Kruskal-Wallis tests, as appropriate.

Models of logistic binomial regression were built using relapse (yes or no) as dependent variable and the predictors having a p value less than 0.3 in univariate analysis.

A p≤0.05 was considered statistically significant.

Statistical analyses were performed with SPSS (SPSS Inc., Chicago, IL) and Analyse-it™ (Analyse-it Software, Ltd., Leeds, UK) packages.

## Results

Complete data were available for 100 patients who were followed for a median period of 3.2 [0.1; 5.5] years.

**Clinical characteristics at presentation**

In the whole cohort, the median age was 61.6 years and there was a slight female preponderance (52%). First symptoms emerged in cold seasons (autumn and winter) in most of cases (62%) and the median time to diagnosis was 2 months, without differences between ANCA groups (**Table 1**).

The clinical condition at presentation was severe (high inflammation, median BVAS 16 and BVAS was over 21 in one quarter of patients), mostly because of general, kidney and lung scores (**Table 1**).

Male gender and younger age were more prevalent among PR3-ANCA patients. PR3-ANCA patients had higher BVAS scores than those in MPA-ANCA group. BVAS scores in patients with N-ANCA were closer to those with MPO-ANCA than to those with PR3-ANCA.

Although the prevalence of kidney, general and chest symptoms was similarly high in all ANCA group, ear, nose and throat (ENT), skin and eye symptoms were more frequent in PR3-ANCA group and respiratory failure was numerically higher in PR3-ANCA (**Table 1**).

The kidney damage was notable in this cohort: median serum creatinine was over 5.66mg/dL in 52% of cases and dialysis was needed in 17% at presentation. Kidney damage was more acute in PR3-ANCA than in MPO-ANCA and N-ANCA, as reflected by the greater prevalence of oliguria (29 vs. 9 and 28%) and the proportion of patients needing dialysis at presentation (38 vs. 11 and 12%). Moreover, hematuria was higher, more frequently macroscopic (71 vs. 27 and 44%) and more frequently associated with red blood cells casts (70 vs. 21 and 30%) (**Table 1**).

**Table 1:** Patients’ characteristics at presentation

Characteristics	All	MPO-ANCA (n=57)	PR3-ANCA (n=24)	N-ANCA (n=19)	Sig.*
Gender (% M)	48%	39%	79%	37%	0.002
Age (years)	61.6 [53.7; 68.4]	62.9 [55.2; 69.7]	56.2 [41.7; 61.7]	66.0 [60.9; 74.5]	0.001
Age >65 years (%)	38%	44%	8%	58%	0.002
Season (winter and autumn; %)	62%	63%	57%	64%	0.58
Time to diagnosis (days)	60 [30; 130]	90 [50; 180]	60 [30; 90]	60 [30; 153]	0.19
BVAS					
- General	3.0 [2.0; 3.0]	3.0 [2.0; 3.0]	3.0 [2.0; 3.0]	3.0 [2.0; 3.0]	0.65
- ENT	0.0 [0.0; 0.0]	0.0 [0.0; 0.0]	0.0 [0.0; 0.0]	0.0 [0.0; 0.0]	0.003
- Chest	0.0 [0.0; 6.0]	0 [0; 5]	4 [0; 6]	0 [0; 6]	0.17
- Kidney	12.0 [12.0; 12.0]	12.0 [12.0; 12.0]	12.0 (12.0; 12.0]	12.0 [12.0; 12.0]	0.24
- Overall	16.0 [15; 21]	15 [14; 19]	21 [15; 23]	16 [15; 20]	0.02
Inflammation					
Hemoglobin (g/dL)	8.4 [7.2; 9.7]	8.4 [7.3; 9.8]	7.6 [6.0; 9.3]	9.0 [7.3; 9.8]	0.08
White blood cells (per mm3)	9,800 [6,867; 13,900]	9,600 [6,700; 12,525]	11,250 [7,800; 15,000]	9,600 [7,033; 12,900]	0.33
Platelets (thousands/mm3)	313 [241; 417]	315 [246; 413]	345 [263; 500]	260 [189; 372]	0.04
ESR (mm/1h)	97 [63; 120]	52 [92; 110]	81 [100; 136]	61 [92; 122]	0.16
Fibrinogen (mg/dL)	620 [517; 798]	640 [514; 800]	672 [541; 800]	612 [489; 714]	0.6
Serum albumin (g/dL)	3.5 [3.1; 3.9]	3.5 [3.1; 3.9]	3.4 [2.9; 3.7]	3.6 [3.0; 4.0]	0.5
Kidney damage					
Oliguria (<800mL/day; %)	18%	9%	29%	28%	0.04
Creatinine (mg/dL)	5.7 [3.1; 8.1]	5.0 [3.0; 7.4]	6.9 [3.7; 9.6]	5.8 [3.9; 8.5]	0.36
Temporary dialysis (%)	17%	11%	38%	12%	0.01
Proteinuria (g/24h)	0.8 [0.5; 2.0]	0.8 [0.5; 2.7]	1.0 [0.4; 1.5]	0.7 [0.4; 1.2]	0.54
Hematuria (cells/mm3)	280 [187; 992]	210 [133; 498]	680 [278; 1.258]	450 [188; 1,000]	0.006
Macroscopic hematuria (%)	41%	27%	71%	44%	0.001
Red blood cell casts (%)	34%	21%	70%	30%	0.002
* MPO-ANCA vs. PR3-ANCA and N-ANCA					
Abbrevations: M: masculine; BVAS: Birmingham vasculitis activity score; ENT: ear, nose, throat; ESR: erythrocyte sedimentation rate.					
Note. Results are presented as percentage or median and quartiles [1; 3].					

**Relapses**

Fifty eight patients (58%) responded to therapy in 3 [0.1; 4.4] months. Only 8 (13.8%) patients relapsed: one in the lung and seven in the kidney. The median time to relapse was 11.3 [9.2; 19.9] months. 5 relapses were in the first year, 2 in the second year and only one in the third year. None of the patients had more than one relapse.

In univariate analysis, none of the investigated parameters allowed differentiating patients who relapsed from those who did not, except higher hematuria in those who relapsed (**Table 2**). In a model of logistic regression which correctly predicted the relapse in 83% of cases, higher ESR and hematuria were the independent predictors, but only hematuria was a significant predictor (**Table 3**).

**Table 2:** Characteristics of patients who relapsed

Parameter	Relapse		Sig.
	Yes	No	
Number	8	50	
General			
Gender (% M)	50	48	0.75
Age (years)	63.3 [51.3; 67.9]	65 [54.3; 72.9]	0.54
Vasculitis			
MPO-ANCA	37%	62%	
PR3-ANCA	37%	22%	0.38
N-ANCA	26%	16%	
BVAS	16 [15; 21]	17 [14-21]	0.37
Number of affected organs	2 [1; 3]	2 [1; 2]	0.65
Inflammation			
WBC (per mm3)	9,950 [7,200-15,058]	9800 [8,200-15,000)	0.74
Platelets (thousands/mm3)	371±116	435±176	0.17
Hemoglobin (g/dL)	8.2 [7.6; 9.4]	9 [7.7; 9.9]	0.28
ESR (mm/1h)	101 [96; 120]	97 [64; 120]	0.24
Fibrinogen (mg/dL)	748±101	690±181	0.25
Serum Albumin (g/dL)	3.5 (3.2-4.0)	3.8 [3.2; 3.4]	0.71
Kidney involvement			
Proteinuria (g/day)	1.5 [0.5; 2.5]	0.6 [0.3; 1]	0.15
Hematuria (cells per mm3)	935 [298; 1592]	210 [159; 290]	0.01
Serum creatinine (mg/dL)	4.1 [3.6; 7.2]	4.7 [2.9; 6.4]	0.78
Abbreviations: M: masculine; BVAS: Birmingham vasculitis activity score; WBC – white blood cells; ESR – erythrocytes sedimentation rate.			
Note. Results are presented as percentage or median and quartiles [1; 3] or mean ± SD as appropriate.			

**Table 3:** Baseline characteristics of young patients

	B	Exp(B)	95%CI		Sig.
Ln (ESR)	2.50	12.24	0.63	236.75	0.10
Ln (Hematuria)	1.15	3.15	1.36	7.33	0.008
Constant	-19.85	0.00	-	-	0.02
Cox & Snell R Square0.23; p=0.03; Hosmer and Lemeshow Test 0.78					

## Discussion

In our cohort, the median age was 61.6 years and gender distribution was balanced, as was reported in other series [**3**-**7**,**9**,**10**,**14**]. The majority of cases were diagnosed in cold seasons, which suggest an association with upper respiratory tract infections as trigger of autoimmunity [**19**,**20**]. As in other studies, the diagnosis was delayed (2 months) [**14**,**21**]. As the first symptoms were general and unspecific, the patients were frequently misdiagnosed, i.e. renal colic, pyelonephritis, cancer or tuberculosis. MPO-ANCA positivity was predominant, which supports the observed South to North gradient of MPO distribution in Europe [**22**].

As in other series of ANCA vasculitis with severe kidney involvement [**5**,**14**,**23**,**24**,**25**], our patients had severe systemic and kidney disease at presentation (median BVAS 16 and median creatinine 5.7 mg/dL; 17% of patients needed dialysis at presentation) and responded in a lower proportion to induction therapy.

There were some difference in presentation according to ANCA specificity. PR3 patients were younger, more frequent males but, in contrast with other studies, they presented with more severe kidney disease than MPO-ANCA patients [**10**,**24**,**25**]. Possible explanation of this discrepancy could be the faster pre-treatment deterioration of kidney function in PR3-ANCA patients, as suggested by Franssen et al [**26**] and the late referral to the nephrologist due to misdiagnosis.

The relapse rate was lower in our patients than in other cohorts (14% vs. 19-56%). Kidney involvement appeared to be “protective” for relapse in cohorts which included renal and non-renal patients [**16**], as the severity of kidney disease was inversely associated with the risk of relapse. Stegeman et al found an almost three times higher risk of relapse (adjusted relative risk, 2.94; 95%CI 1.27 to 6.67) in patients with GPA and mild kidney disease as in those with more severe kidney involvement (creatinine clearance <60 ml/min) [**27**]. The same positive correlation between kidney function and relapses was also found in MPO-ANCA patients with pauci-immune glomerulonephritis [**28**]. Even if the patients on chronic dialysis still relapse and need immunosuppressive treatment, the relapse rate is significantly lower than in the pre-dialysis period [**29**]. One explanation could be the immune system suppression with advanced chronic kidney disease. In our cohort, renal function was not related to relapse, probably because the kidney disease was uniformly severe: median serum creatinine 4.1 and 5.9 mg/dl in those who relapsed or not, respectively.

However, our relapse rate (14%) was substantially lower as compared to reports in patients with similar severity of kidney disease (42%, 59%) [**6**,**10**]. Possible explanations are the routine long term maintenance therapy and the predominant MPO-ANCA serology in our cohort,as the higher relapse rates were almost unanimously reported in PR3-ANCA patients [**6**,**8**,**14**]. However, in univariate analyze ANCA specificity did not influenced the rate of relapse.

Only hematuria at presentation differentiated relapsing patients. In contrast with serum creatinine and proteinuria which can not differentiate between acute or chronic injury, hematuria is associated only with activity. With other words, patients which presents with more activity in the kidney are more prone to relapse after remission.

Similar to other reports, most of relapses were seen in patients on maintenance therapy [**3**,**9**]. This underlines the necessity of a better alternative to the standard maintenance regimen with azathioprine and low dose prednisone. Indeed, two very recent studies, one observational and one randomized, assessed the efficacy and the safety profile of rituximab versus azathioprine for maintenance of remission in ANCA vasculitis [**29**,**30**]. They found significantly lower relapse rates in patients treated with rituximab (5%) and improved survival to that seen in the general population, which also highlightsthe importance of relapses for patients’ outcome [**29**,**30**].

There are some limits of our study. The number of participants and the number of events wererelatively low and the period of observation rather short, which limit the statistical power didn’t allow us to perform more sophisticated analyses. ANCA were assessed by indirect immunofluorescence and/or by ELISA, and discrepancies between results obtained by these methods were described.

In conclusion, in our patients with ANCA vasculitis with severe kidney involvement, the relapse rate is low and hematuria but not ANCA specificity or clinical presentation allows the prediction of relapse.

**Source of funding**

This study and the authors had no financial support.

**Disclosures**

None to declare.
